# Membrane Bound CRT Fragment Accelerates Tumor Growth of Melanoma B16 Cell In Vivo through Promoting M2 Polarization via TLR4

**DOI:** 10.1155/2022/4626813

**Published:** 2022-10-06

**Authors:** Hong-Min Wang, Zhe Zhou, Jie Miao, Bo Zhu, Xiao-Qiu Dai, Qiao Zhong, Fang-Yuan Gong, Xiao-Ming Gao

**Affiliations:** ^1^Institute of Biology and Medical Sciences, School of Biology and Basic Medical Sciences, Soochow University, Suzhou 215123, China; ^2^Department of Laboratory Medicine, Gusu School, The Affiliated Suzhou Hospital of Nanjing Medical University, Suzhou 215002, China; ^3^Key Laboratory of Systemic Biomedical Study, Suzhou 215123, China

## Abstract

Calreticulin (CRT) is a major calcium-binding luminal resident protein on the endoplasmic reticulum that can also be released extracellular as well as anchored on surface of cells. Previously, we demonstrated that soluble recombinant CRT fragment 39-272 (CRT/39-272) exhibited potent immunostimulatory effects as well as immunoregulation effects on immune cells. Here, we constructed stable B16 melanoma cell lines expressing recombinant CRT/39-272 on the membrane (B16-tmCRT/39-272) to investigate the roles of cell surface CRT on tumor progression. We found that B16-tmCRT/39-272 cells subcutaneously inoculated into C57BL/6 mice exhibited stronger tumorigenicity than the B16-EGFP control cells. The tumor associated macrophages infiltrated in tumors were mainly M2 phenotype. Regulatory *T* cells (Tregs) were also expanded more in bearing mice. Consistent with the *in vivo* results, B16-tmCRT/39-272 promoted macrophage polarization toward F4/80^+^CD206^+^ M2 macrophages and promoted transforming growth factor beta (TGF-*β*) secretion *in vitro*, which could promote naïve CD4^+^*T* cell differentiation into Tregs. These results imply that the tmCRT/39-272 could accelerate tumor development by enhancing M2 macrophage polarization to induce TGF-*β* secretion, and then promoted Treg differentiation in the tumor microenvironment. Our data may provide useful clues for better understanding of the potentiating roles of CRT in tumorigenesis.

## 1. Introduction

Calreticulin (CRT) is a calcium (Ca^2+^)-binding chaperone protein predominantly localized in the endoplasmic reticulum (ER). CRT consists of three domains: a lectin-like N-domain, a proline-rich P-domain, and a Ca^2+^-binding C-domain, and it contains an N-terminal amino acid signal sequence and a C-terminal KDEL ER retrieval signal sequence [1, 2]. CRT is believed to play important roles in various cellular processes, such as modulating cell adhesion, migration, phagocytosis of apoptotic cells, and immune response [3–7]. As a tumor-associated antigen (TAA), CRT triggers a specific antitumor immune response. On the other hand, CRT is highly expressed in different cancer cells or tumor tissues [8–10]. Therefore, CRT plays dual roles in tumor development and progression and is one of the key molecules not only in the tumorigenesis process, but also in tumor immune surveillance and clearance.

CRT is a multifunctional protein expressed not only in the cytoplasm but also in the extracellular matrix and on the cellular surface [3, 11–16]. CRT typically resides in the ER where it is retained through its C-terminal KDEL retention signal sequence, typically not secreted into the extracellular matrix or attached to the membrane [1]. However, it can be released to the cell surface under specific conditions, such as cell damage by cytotoxic drugs or inflammation, or apoptosis by *γ*-irradiation or chemotherapeutic agents. Cell surface CRT is considered an “eat-me” signal that contributes to the phagocytic uptake of cancer cells and dying cells by the immune system [3, 15, 17, 18], which is recognized by phagocyte CD91 (CRT receptor) and mediates phagocyte recognition and clearance of apoptotic tumor cells [19–22]. Furthermore, accumulated evidence has indicated that CRT is associated with tumor formation and progression. Recent studies have indicated that CRT was expressed on the surface of tumor-associated macrophages (TAMs) and plays an important role in mediating adjacent tumor cell recognition and phagocytosis [20, 23].

Previously, we determined that the recombinant CRT fragment 39-272 (CRT/39-272), which lacks the C-terminus and KDEL motif, exhibited potent immunostimulatory activity and strong adjuvanticity, and soluble CRT/39-272 (sCRT/39-272) promoted tumor malignancy through TLR4- and S100A8/9-mediated myeloid-derived suppressor cell (MDSC) differentiation and recruitment [24]. However, it remains unknown whether transmembrane CRT/39-272 (tmCRT/39-272) could also influence tumorigenesis *in vivo*. In the present study, we will construct B16 cell line stably expressing tmCRT/39-272 (B16-tmCRT/39-272) and explore tumor growth and immune response of B16-tmCRT/39-272.

## 2. Materials and Methods

### 2.1. Mice

Female C57BL/6 (H-2b) mice and *TLR4* knockout (TLR4-KO) mice of C57BL/6 background aged 6-8 weeks were purchased from the Model Animal Research Center of Nanjing University (Nanjing, China). Foxp3-GFP mice of C57BL/6 background were generously provided by Dr. J. Zhang (Soochow University, Suzhou, China). All animals were maintained under specific pathogen-free conditions and all experiments were performed in accordance with procedures approved by the Animal Care and Use Committee of Soochow University.

### 2.2. Cell Culture

The B16 melanoma cell line was purchased from the Cell Bank of the Chinese Academy of Sciences (Shanghai, China). The cells were retrovirally transfected with mouse CRT/39-272 complementary DNA (cDNA), a leading sequence, and a transmembrane domain at the N-terminal (B16-tmCRT/39-272) or with empty vector (B16-EGFP) using LV5-CRT-Puro lentivirus vector. Transfection efficiency was estimated by enhanced green fluorescence protein (EGFP) fluorescence intensity and CRT expression in the transduced cells. Stable cell lines underwent fluorescence-activate cell sorting (FACS) or were screened by culture medium containing 5 *μ*g/mL puromycin (Sigma, St. Louis, USA). The B16, B16-EGFP, and B16-tmCRT/39-272 cells were cultured in Dulbecco's modified Eagle's medium (DMEM) complemented with 10% fetal bovine serum (FBS, Hyclone, Utah, USA) and 1% penicillin-streptomycin. C57BL/6 mouse bone marrow cells were cultured in 30 ng/mL M-CSF conditioned R10 medium: RPMI 1640 medium supplemented with 10% FBS and 1% penicillin-streptomycin. All cells were incubated at 37°C with 5% CO_2_ in a humidified cell culture incubator.

### 2.3. MTT Assay

Cell viability was evaluated by the 3-(4,5-dimethylthiazol-2-yl)-2,5-diphenyltetrazolium bromide (MTT) reduction method. B16-EGFP and B16-tmCRT/39-272 cells were plated in 96-well culture plates at 5 × 10^3^ cells per well in a total volume of 200 *μ*L DMEM, cultured for 0-4 d, and analyzed using an MTT kit according to the manufacturer's instructions (Sigma, St. Louis, Mo). Briefly, the cells were stained with 20 *μ*L MTT (5 mg/mL) for 4 h. The precipitated formazan was dissolved in dimethyl sulfoxide and the optical density (OD) was measured at 545 nm using a spectrometer (Spark, Tecan, CH). The relationship between absorbance and cell number was determined using a standard curve that had been established for a known cell number from previous incubation with MTT. All experiments were performed in triplicate.

### 2.4. Cell Adhesion Assay

The cells were counted with a hemocytometer to a concentration of 2.5 × 10^6^ cells/mL and 100 *μ*L culture medium containing the cells was added to each well of Matrigel (10 *μ*g/mL)-precoated 96-well flat-bottomed plates and incubated at 4°C overnight. The next day, the plates were incubated for 20 min, 40 min, and 60 min at 37°C in 5% CO_2_. Then, nonadherent cells were removed by washing with phosphate-buffered saline (PBS). The absorbance of the attached cells was measured using the MTT assay at 570 nm wavelength in a spectrometer.

### 2.5. Wound Healing Assay

A total of 5 × 10^6^ cells were seeded on 6-well plates in triplicate. A wound was scratched in the confluent cell monolayer with a 200-*μ*L pipette tip. Detached cells were washed with PBS and cultured in fresh serum-free DMEM. For each well, photographs were captured at 0 h (immediately after the scratch), 12 h, and 24 h. The images were recorded and the wound gap area was measured by ImageJ. Wound Closure (%) was calculated as following: [gap area (*T*_0_ − *T*)/gap area *T*_0_] × 100%, where *T* is the treatment time and *T*_0_ is when the wound was created.

### 2.6. Flow Cytometry

The cells were collected, resuspended in binding buffer, stained with fluorescence-labeled antibodies against mouse CRT, CD3, CD4, CD8, NK1.1, CD11c, Gr-1, CD11b, F4/80, CD206, or tumor necrosis factor alpha (TNF-*α*) (alone or in different combinations) for 30 min at 4°C, followed by washing and resuspension in fixation buffer. The stained cells were analyzed using a fluorescence-activated cell sorter (Attune NxT, Life Technologies, CA, USA). The FACS data were analyzed using FlowJo.

### 2.7. Tumor Cell Inoculation

Female C57BL/6 or TLR4-KO C57BL/6 mice were subcutaneously (s.c.) injected with B16-EGFP or B16-tmCRT/39-272 cells (5 × 10^5^/100 *μ*L/mouse). The tumor size and volume were measured every 2 or 3 days. The diameter of solid tumors at the injection sites was evaluated using vernier calipers and the tumor volume was estimated as follows: 0.5 × width^2^ × length [25–27]. At the end of the 2-3 weeks, the tumors were removed from the mice and analyzed.

### 2.8. Immunofluorescence Staining

B16-EGFP and B16-tmCRT/39-272 cells were cultured in a Poly-L-lysine-coated 8-well chamber slide system for 4 h, fixed with 4% paraformaldehyde (PFA), and stained with anti-CRT antibody (cell membrane) and DAPI (nucleus). B16-EGFP and B16-tmCRT/39-272 solid tumor tissues from the C57BL/6 mice were cut into 5-*μ*m frozen sections and fixed in cold 4% PFA at 4°C for 30 min. The sections were stained by incubation with 1% bovine serum albumin in PBS at room temperature for 30 min, followed by incubation with anti-CD206 overnight at 4°C. Finally, the sections were counterstained with 1 mg/mL DAPI. The stained cells or sections were analyzed by confocal scanning laser microscopy.

### 2.9. In Vitro Differentiation of CD4-Positive T Cells

To prepare CD4-positive T (CD4^+^ T) cells, Foxp3-GFP C57BL/6 mice spleens were gently disaggregated by pressing with the flat surface of a syringe plunger against a stainless steel sieve (200 mesh) and treated with red blood cell lysis buffer. Single cell suspensions were enriched using MojoSort™ Mouse CD4 T Cell Isolation Kit (BioLegend, San Diego, USA). Briefly, the spleen cells were resuspended in MojoSort™ buffer, and then the cell suspension was incubated with biotin-antibody cocktail on ice for 15 min, followed by incubation with streptavidin nanobeads. The CD4^+^ T cells were purified using magnetic bead depletion of other cell populations in the spleen. Flow cytometry demonstrated that the purity of the resultant CD4^+^*T* cells was >90%.

To differentiate splenic CD4^+^ T cells, plates were precoated with CD3 antibody (1 *μ*g/mL) and incubated at 4°C overnight. The cells (1 × 10^5^/well) were seeded in 96-well round (*U*)-bottomed plates, to which were added CD28 antibody (1 *μ*g/mL), the recombinant cytokines IL-2 (5 ng/mL) and TGF-*β* (5 ng/mL), and the B16-EGFP or B16-tmCRT/39-272 suspensions.

### 2.10. Preparation of Supernatant

C57BL/6 mouse bone marrow cells were cultured in 30 ng/mL M-CSF conditioned R10 medium for 7 days, followed by the addition of B16-EGFP or B16-tmCRT/39-272 cells treated with *γ*-ray (6500 cGy). After 24 h incubation with 5% CO_2_ at 37°C, the cell culture supernatants were collected using 0.22-*μ*m filters (Millipore, MA, USA) and stored at ­80°C.

### 2.11. Enzyme-Linked Immunosorbent Assay (ELISA)

For quantitative analysis of TGF-*β* in the cell culture supernatant, ELISA kits were used following the manufacturer's instruction (eBioscience, CA, USA). Antimouse TGF-*β* antibody was precoated onto the microwells. Following incubation, unbound biological components were removed during a wash step with 1× wash buffer. Then, 100 *μ*L diluted sera and detection antibodies were added to the well, followed by 90 min incubation at room temperature. After five washes with 1× wash buffer, the plates were incubated with horseradish peroxidase-streptavidin for 30 min at room temperature. The reaction was developed with 100 *μ*L o-phenylenediamine for 5 min and stopped with 100 *μ*L 2 M H_2_SO_4_. The OD of the wells was measured at 492 nm using an ELISA spectrophotometer.

### 2.12. Macrophage Depletion

Female C57BL/6 mice were s.c. injected with B16-EGFP or B16-tmCRT/39-272 cells (5 × 10^5^/100 *μ*L/mouse). To deplete macrophages, 100 *μ*L clodronate liposomes (Lipo-Clod), or control liposomes (Lipo-PBS) in 1 mg/mL was i.p. injected using a 26-gauge needle. The liposomes were injected every 3 days after tumor cell injection to deplete the infiltrating macrophages. On day 12 of the experiment, the ascites was collected and the effect of macrophage depletion was determined using anti-CD11b and anti-F4/80 antibody staining.

### 2.13. Statistical Analysis

All experiments were repeated at least three times and the results are expressed as the mean ± standard deviation of the mean (SD). The statistical analysis was performed using Student's *t*-test among groups with GraphPad Prism 5.0. A *P*value <0.05 was considered significant in all experiments.

## 3. Results

### 3.1. Recombinant tmCRT/39-272 Promoted Tumorigenicity of B16 Cells In Vivo

CRT was reported to be anchored on the membrane of tumor cells under immunogenic cell death which could inhibit tumorigenesis. However, we found that CRT/39-272 could induce accumulation of MDSCs to promote tumorigenesis. To explore the effect of cell surface CRT/39-272 on tumorigenesis, we constructed tumor cells expressing transmembrane CRT/39-272 (tmCRT/39-272).

**Figure 1:**
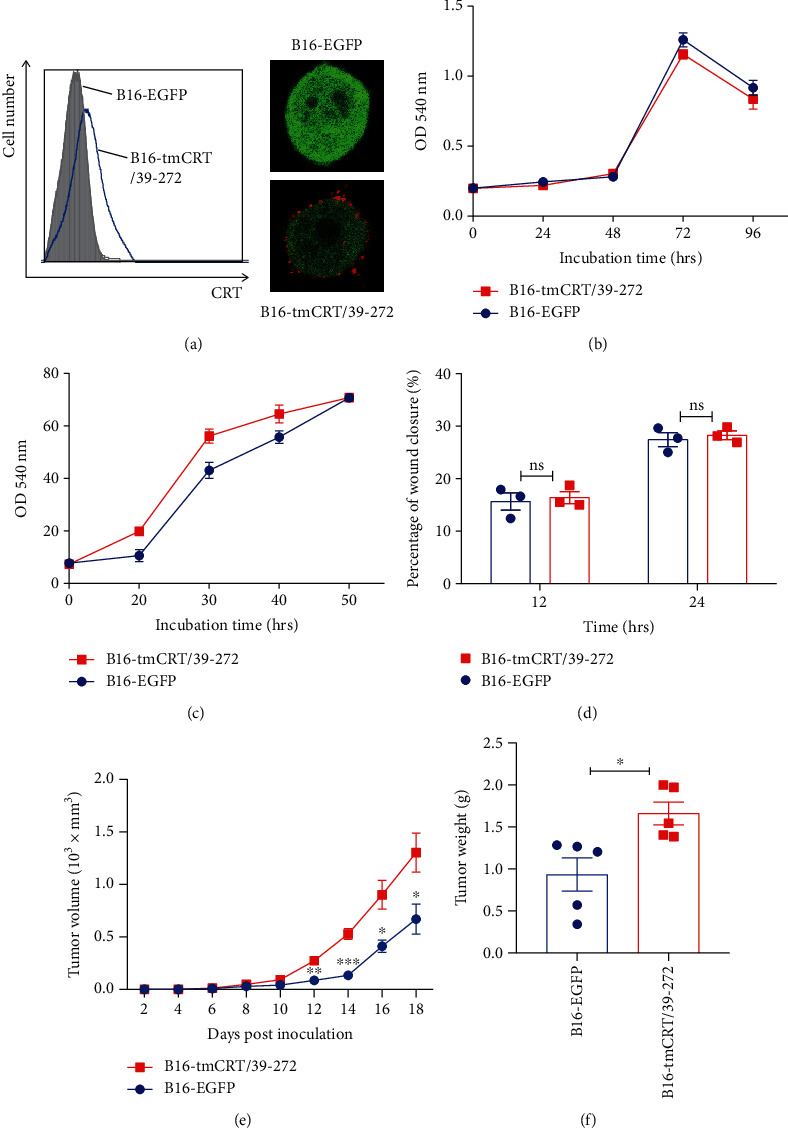
Construction and characterization of the B16-EGFP and B16-tmCRT/39-272 cell lines. (a) Surface expression of CRT on B16-EGFP and B16-tmCRT/39-272 cells were examined by FACS and confocal laser scanning microscopy. (b) MTT assay of B16-EGFP and B16-tmCRT/39-272 cell proliferation. (c) Cell adhesion assay of B16-EGFP and B16-tmCRT/39-272 cells post-scratch. (d) Wound healing assay for examining B16-EGFP and B16-tmCRT/39-272 cell migration. The width of the wound was measured 12 h and 24 h after wounding. (e and f) Female C57BL/6 mice (*n* = 5 per group) were s.c. injected with B16-EGFP or B16-tmCRT/39-272 cells (5 × 105/100 *μ*l/mouse). The diameter of solid tumors at the injection sites was evaluated every 2 days thereafter for 18 days. (e) Tumor growth curves. (f) Tumor weights. The experiments were repeated three times. ^∗^*P* < 0.05, ^∗∗^*P* <0.01, ^∗∗∗^*P* < 0.001, ns: not significant.

We constructed a stable murine B16 melanoma cell line expressing recombinant tmCRT/39-272 (B16-tmCRT/39-272), with recombinant EGFP (rEGFP) as a coexpression marker. B16 cells expressing rEGFP (B16-EGFP) alone were included as control. As shown in Figure 1(a), CRT/39-272 could be detected by anti-CRT on the surface. There was no difference between these two cell lines in terms of spontaneous proliferation, adhesion to fibrinogen-coated surface, and wound healing assays (Figures 1(b)–1(d)), indicating that tmCRT39-272 expression per se did not have direct effects on B16 malignancy. When s.c. inoculated B16-tmCRT/39-272 and B16-EGFP cells into C57BL/6 mice, however, B16-tmCRT/39-272 cells grew significantly faster than B16-EGFP control cells (Figure 1(e)). At the end of the experiment, i.e., day 18 postinjection, B16-tmCRT/39-272 tumors were significantly larger in weight as well (Figure 1(f)). These results indicate that recombinant tmCRT/39-272 promoted B16 cell tumorigenesis *in vivo*, which is exactly the reverse anti-tumorigenesis effect of transmembrane full length CRT.

### 3.2. Recombinant tmCRT/39-272 Promoted Macrophage Differentiation into the M2-like Phenotype Both In Vitro and In Vivo

**Figure 2:**
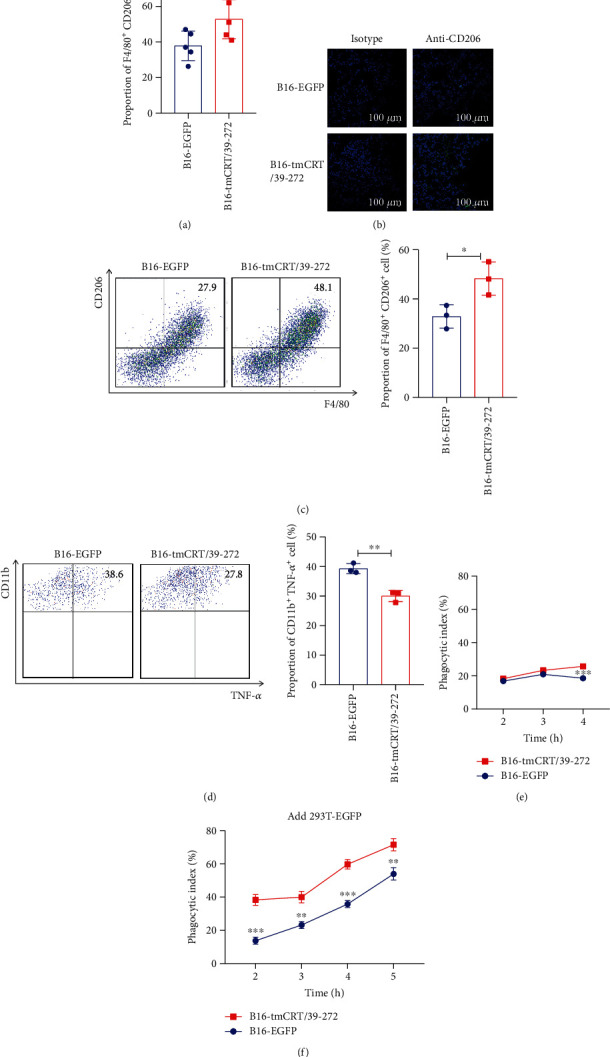
tmCRT/39-272 enhanced macrophage phagocytosis and M2 macrophage polarization. (a) Female C57BL/6 mice (*n* = 5) were s.c. injected with B16-EGFP or B16-tmCRT/39-272 cells (5 × 105/100 *μ*L/mouse) and sacrificed 18 days postinoculation to obtain the solid tumors. B16-EGFP and B16-tmCRT/39-272 tumor tissues were digested with collagenase Ⅳ (1 mg/mL) and DNase Ⅰ (0.05 mg/mL). Single cells were filtered through a 70-*μ*m cell strainer and stained with fluorescence-labeled anti-F4/80 and anti-CD206 antibodies. The percentage of F4/80^+^CD206^+^ M2 macrophages among the tumor cells was analyzed by FACS. (b) Immunofluorescence staining of tumor tissues with FITC-labeled anti-CD206 antibody (green) and DAPI (blue). Images were acquired by laser-scanning confocal microscopy. (c and d) FACS dot plots and the statistical results of percentage of F4/80^+^CD206^+^ cells and CD11b^+^TNF-*α*^+^ cells from C57BL/6 mouse bone marrow cells analyzed by flow cytometry. (e) B16-EGFP or B16-tmCRT/39-272 cells were labeled with CFSE and cocultured with macrophages at a ratio of 3:1 for 2 h, 3 h, and 4 h. The cells were stained with fluorescence-labeled antibody against anti-CD11b and the phagocytic capability of the macrophages was analyzed by flow cytometry. (f) 293T-EGFP cells (labeled with dye) were added to macrophage cocultured with B16-EGFP or B16-tmCRT/39-272 cells. The cell ratio was 1:1:3 (macrophage:293T-EGFP:B16-EGFP or B16-tmCRT/39-272). At 2 h, 3 h, 4 h, and 5 h, the cells were collected for staining with fluorescence-labeled anti-CD11b antibody and the phagocytic capability of the macrophages on 293T-EGFP cells was assessed by flow cytometry. The experiments were repeated three times.^∗^*P* < 0.05, ^∗∗^*P* < 0.01, ^∗∗∗^*P* < 0.001.

Although the B16-tmCRT/39-272 solid tumors harvested from C57BL/6 mice at 18 days postinoculation were larger than that of the B16-EGFP group, the tumor cell-derived tmCRT/39-272 could not directly promote B16 cell proliferation (Figure 1(b)), so the shaped of cell populations in tumor environment could be the mechanism to explain its malignancy-enhancing effect. Tumor infiltrated immune cells from B16-EGFP and B16-tmCRT/39-272 bearing mice were analyzed by FACS. The proportions of Dendritic cells, MDSCs, natural killer cells, and T cells in tumor tissues, showed no significant difference between the two groups (Figure S1). Macrophages are particularly abundant among infiltrating immune cells in solid tumor tissues, which show immunoregulatory effect in tumor environment to promote tumorigenesis [28]. The macrophages in the tumor environment (TAM) are M2 phenotype expressing high level of CD206. As shown in Figure 2(a), the proportion of M2-like macrophages was higher in the B16-tmCRT/39-272 group than in the B16-EGFP group. Furthermore, immunofluorescence staining analysis for M2 macrophages among the disseminated cells from the solid tumor tissues further confirmed the enrichment of M2 macrophages in B16-tmCRT/39-272 tumors (Figure 2(b)).

In order to explore whether B16-tmCRT/39-272 could enhance M2 macrophage differentiation directly, the phenotype of macrophages generated from bone marrow (BMDMs) which were cocultured with irradiated B16-tmCRT/39-272 or B16-EGFP was determined. The F4/80^+^CD206^+^ cell population of B16-tmCRT/39-272 group accounted for 48.3%, significantly higher than that of the B16-EGFP control group (32.9%) (Figure 2(c)), and the proportion of CD11b^+^TNF-*α*^+^ cells in the B16-tmCRT/39-272 group was significantly lower than that of the B16-EGFP group (30.0 vs. 39.3%) (Figure 2(d)). Together, these results suggested that recombinant tmCRT/39-272 polarized macrophages into the M2 phenotype to promote tumorigenesis.

As reported that tmCRT could act as an “eat me” signal to enhance phagocytosis of dead tumor cells [3, 18]. M2 phenotype macrophages showed greater phagocytic capacity compared with M1 macrophages [29]. Thus, the enhancement of phagocytosis by tmCRT/39-272 was also evaluated. BMDMs were cocultured with carboxyfluorescein succinimidyl ester (CFSE) labeled B16-tmCRT/39-272 or B16-EGFP cells for indicated time. The macrophages exhibited higher phagocytic ability against B16-tmCRT/39-272 cells as compared with the B16-EGFP control cells (Figure 2(e)). Additionally, 293 T cells (labeled with dye) were incubated with macrophages which were precultured with B16-tmCRT/39-272 or B16-EGFP. As shown in Figure 2(f), the phagocytic ability of macrophages incubated with B16-tmCRT/39-272 was stronger than that incubated with B16-EGFP, indicating that tm-CRT/39-272 could enhance the phagocytosis ability of macrophages.

### 3.3. Macrophage Depletion Reduced Tumor Tumorigenesis of tmCRT/39-272 In Vivo

**Figure 3:**
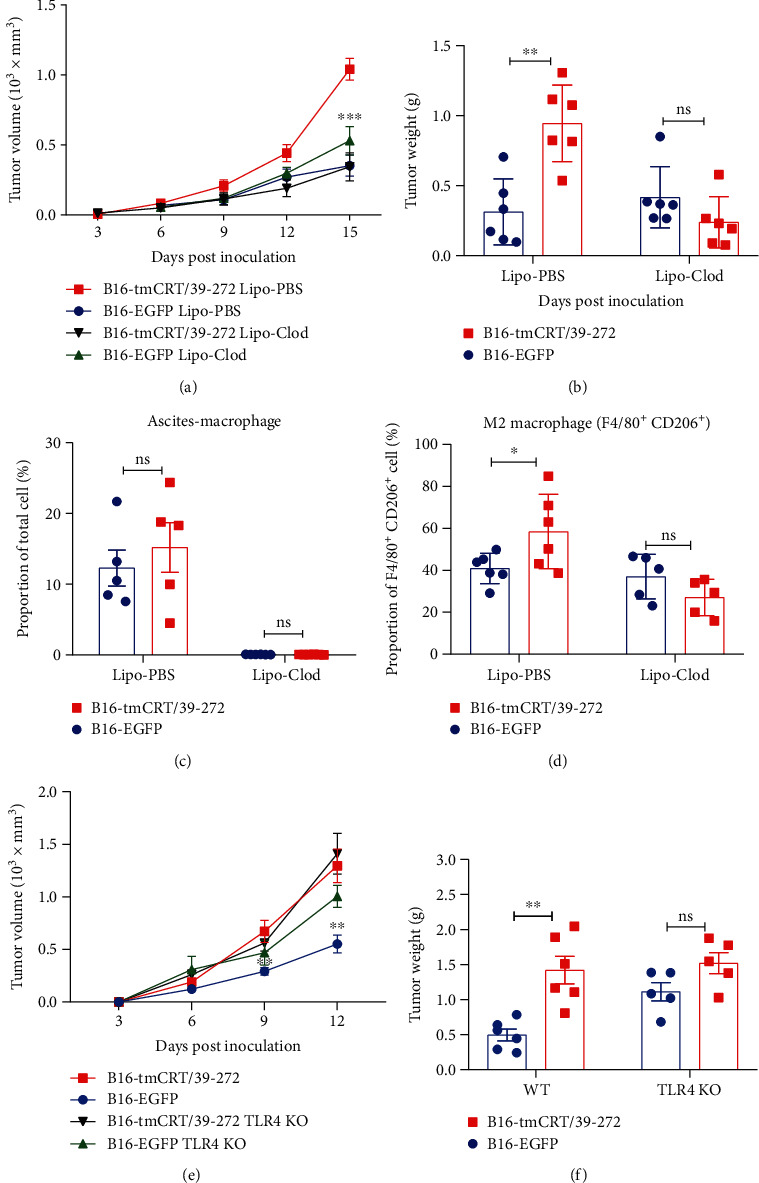
Macrophage depletion or TLR4 KO reduced tumorigenesis and M2 polarization in B16-tmCRT/39-272-inoculated mice. (a–d) Female C57BL/6 mice (*n* = 6) were s.c. injected with B16-EGFP or B16-tmCRT/39-272 cells (5 × 105/100 *μ*L/mouse), then i.p. injected with Lipo-Clod or Lipo-PBS (100 *μ*L/mouse) every 3 days. (a) Tumor growth curves. (b) Tumor weights. (c) Ascites macrophages were collected from the mice 15 days postinoculation. The proportions of total macrophages (CD11b^+^F4/80^+^) were analyzed by FACS. (d) Tumor tissues were collected from mice 15 days postinoculation. The proportions of M2 macrophages (F4/80^+^CD206^+^) were analyzed by FACS. (e and f) Female C57BL/6 (*n* = 6) or TLR4-KO C57BL/6 mice (*n* = 5) were s.c. injected with B16-EGFP or B16-tmCRT/39-272 cells (5 × 10^5^/100 *μ*L/mouse) and the diameter of solid tumors at the injection sites was evaluated every 3 days thereafter. (e) Tumor growth curves. (f) Tumor weights. The experiments were repeated three times. ^∗^*P* < 0.05, ^∗∗^*P* < 0.01, ^∗∗∗^*P* < 0.001, ns: not significant.

In order to determine whether macrophages play important roles in the enhancement of tumorigenesis, clodronate liposome (Lipo-Clod) was employed to deplete macrophages *in vivo*. Tumor bearing mice were treated with Lipo-Clod every 3 days and tumor sizes were evaluated. The average tumor volume and weight in the B16-tmCRT/39-272 group were significantly larger than that of the B16-EGFP group (Lipo-PBS treatment), but there was no significant difference between the two groups after Lipo-Clod injection (Figures 3(a) and 3(b)).

At the end of the experiment, macrophages in ascites were collected and analyzed by flow cytometry, which demonstrated that macrophages were completely diminished after Lipo-Clod treatment as compared with the Lipo-PBS control, indicating that the Lipo-Clod was efficacious (Figure 3(c)). The enrichment of F4/80^+^CD206^+^ cells (M2 macrophages) in B16-tmCRT/39-272 tumor bearing mice was also diminished after Lipo-Clod treatment. (Figure 3(d)). Taken together, these results suggested that M2 macrophages played an important role in tmCRT/39-272-secreting solid tumors *in vivo.*

### 3.4. The Enhancement of Tumorigenesis by tmCRT/39-272 Was Dependent on TLR4

TLR4 were reported to be one of the receptors for soluble CRT. To explore the role of TLR4 in tmCRT/39-272 promoting tumorigenesis, TLR4-KO mice were used to evaluate tumor growth of B16-EGFP or B16-tmCRT/39-272 *in vivo*. WT and TLR4 KO mice were s.c. injected with B16-EGFP or B16-tmCRT/39-272 cells (5 × 10^5^/100 *μ*L/mouse) and solid tumor formation at the injection sites was observed every 3 days. By day 12 postinoculation, the B16-tmCRT/39-272 cells appeared to be much more aggressive than the B16-EGFP control, as the earlier results showed, but there was no significant difference in the weight of the two TLR4-KO mouse groups (Figures 3(e) and 3(f)), indicating that tmCRT/39-272 promoted tumor malignancy through interaction with TLR4.

### 3.5. Treg Differentiation Was Promoted by Enriched M2 Macrophages through TGF*β*

**Figure 4:**
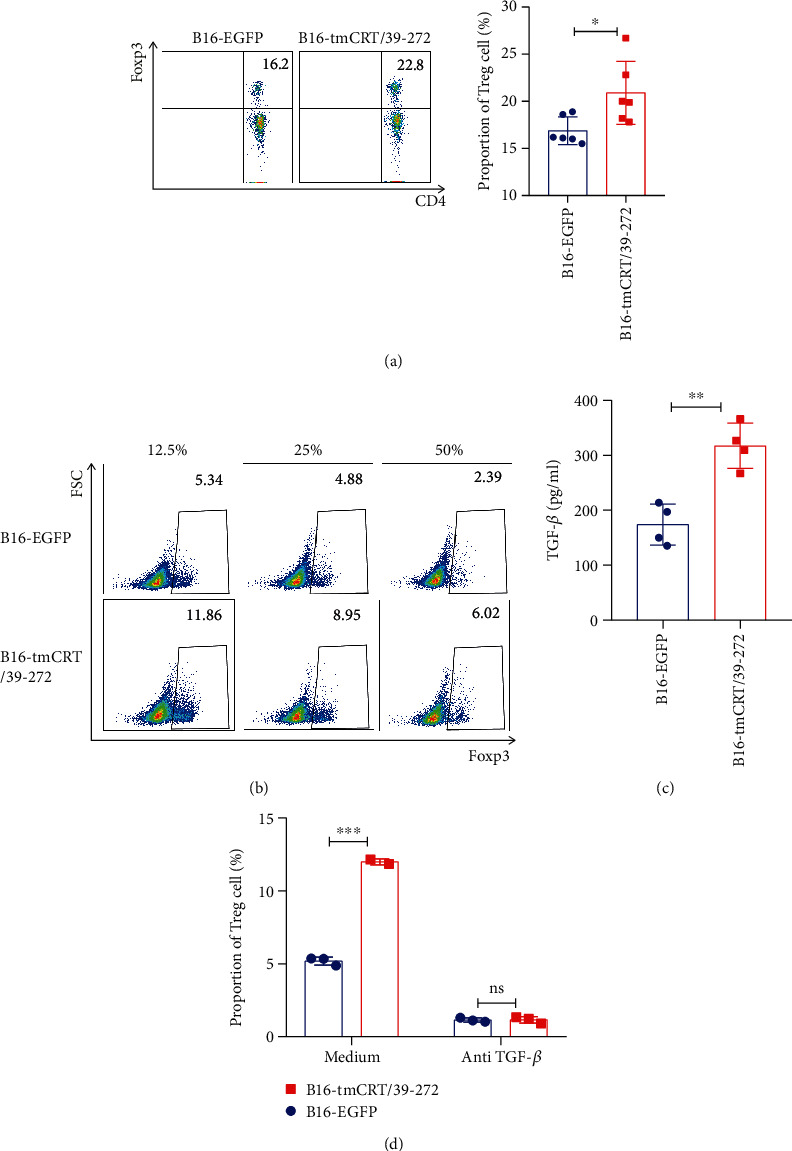
tmCRT/39-272 promoted Treg differentiation through TGF-*β* secretion by M2 macrophages. (a) Female Foxp3-GFP C57BL/6 mice (*n* = 6) were s.c. injected with B16-EGFP or B16-tmCRT/39-272 cells (5 × 105/100 ***μ***L/mouse). Spleen cells were collected from the mice 12 days postinoculation. The proportions of Tregs were analyzed by FACS. FACS dot plots and the statistical results of Tregs are shown in panel. (b) Naïve CD4^+^ T cells purified from the spleens of Foxp3-GFP C57BL/6 mice, different doses (50%, 25%, 12.5%) of B16-EGFP or B16-tmCRT/39-272 cell supernatants were added to the wells for 72 h. Single cells were collected and the populations of Treg analyzed by FACS. (c) ELISA of TGF-*β* in the supernatants of C57BL/6 mouse bone marrow cells cultured in M-CSF conditioned medium, followed by the addition of *γ*-ray-treated B16-EGFP or B16-tmCRT/39-272 cells. (d) B16-EGFP or B16-tmCRT/39-272 cell supernatants (12.5%) were added to the naïve CD4^+^ T cells (purified from the spleens of Foxp3-GFP C57BL/6 mice) and treated with or without TGF-*β*-blocking antibody (10 ng/mL) for 72 h. Single cells were collected and analyzed by FACS. The experiments were repeated three times. ^∗^*P* < 0.05, ^∗∗^*P* < 0.01, ^∗∗∗^*P* < 0.001, ns: not significant.

The percentage of CD4^+^CD25^+^ Tregs in the spleen was significantly higher in the B16-tmCRT/39-272 tumor-bearing mice than in the B16-EGFP controls (16.88% vs 20.9%) (Figure 4(a)), which contributed to enhanced the tumorigenesis of B16-tmCRT/39-272. M2 macrophages could induce Treg differentiation through secreting immune-regulatory cytokines. To assess whether B16-tmCRT/39-272 primed macrophages could promote Treg differentiation directly, Treg differentiation was analyzed after incubation with supernatant from cocultures of B16-tmCRT/39-272 or B16-EGFP control cells and macrophages. As shown in Figure 4(b), compared with the B16-EGFP group, the B16-tmCRT/39-272 group exhibited increased Treg differentiation. Furtherly, immunoregulatory cytokines in the supernatant from cocultures of B16-tmCRT/39-272 or B16-EGFP control cells and macrophages were determined. Among them, TGF-*β* was found to be enriched in the supernatants of B16-tmCRT/39-272 primed macrophages (Figure 4(c)). The addition of anti-TGF-*β* blocking antibody to the samples decreased the Treg differentiation, demonstrating that the anti-TGF-*β* antibody was efficacious (Figure 4(d)). Together, these data demonstrated that the enhanced tumorigenicity of recombinant tmCRT/39-272 was largely dependent on the TGF-*β* secretion by M2 macrophages.

## 4. Discussion

Recent research has demonstrated that CRT is present in most ER, the cell surface, and extracellularly. The multiple functions and properties of CRT are closely related to cell proliferation and transformation, either via inhibitory (antioncogenic) or stimulatory (oncogenic) effects [30]. The N-terminal domain of the CRT, vasostatin is a highly potent endogenous inhibitor of angiogenesis and tumor growth [31–33]. CRT enhances the expression of tumor endothelial adhesion molecules and promotes tumor-specific lymphocyte infiltration.

CRT exposure was critical for immunogenicity in cancer cell death. During cancer cell apoptosis induced by chemotherapeutic agents, CRT could be translocated from the intracellular compartment to the cell membrane, with clustered distribution on apoptotic cells. Concomitantly, cell surface CRT acts as an “eat-me” signal and binds to LRP (CD91) by increasing macrophage phagocytosis and clearance of dead cells [18]. Cell surface CRT also interacts with complement C1q receptor for apoptotic cell uptake and removal [34–39]. On the other hand, evidence from numerous studies has suggested that CRT is highly expressed in different tumor cells and its expression was positively correlated with tumor progression [40, 41]. Therefore, CRT plays contradictory roles in tumor development.

Tumor metastasis/development and progression rely on the mutual interaction of cancer cells and their environment, and form the TME. The TME comprises different cellular components, such as endothelial cells, immune cells, and fibroblasts. These cells can release various cytokines in response to the immune system, thereby facilitating or inhibiting tumor cell proliferation, migration, and survival. Immune cells in the TME consist of granulocytes, lymphocytes, and macrophages [42]. Macrophages are considered the most prominent immune cell type in the TME and can be categorized into M1-like and M2-like subtypes based on their polarization status. M1 macrophages can be activated by the Th1 cytokine interferon *γ* (IFN-*γ*) and exert tumoricidal effects. In contrast, M2 macrophages differentiate in response to Th2 cytokines, e.g., IL-4. As the immunosuppressive cell type, M2 macrophages exhibit anti-inflammatory and protumoral effects [43].

Previously, we reported that the CRT fragment (recombinant sCRT/39-272) promoted tumor malignancy through TLR4- and S100A8/9-mediated MDSC differentiation and recruitment [24]. However, whether tmCRT/39-272 is involved in tumor development has not been well-investigated. In the present study, tmCRT/39-272 accelerated tumor malignancy/progression. However, there was virtually no difference in terms of cell proliferation, adhesion, and migration. The CRT in our system lacked the C-terminal KDEL ER retrieval signal sequence, but the increased intracellular CRT expression could not be concentrated in the ER. Therefore, our results were not inconsistent with previously reported results.

We determined that tmCRT/39-272 promoted the phagocytic ability of macrophages against tumor cells during the initial stages of interaction between macrophages and tumor cells. The results were consistent with that of several prior studies that showed that membrane CRT aided the immune system in eliminating tumor cells. However, contradictory to the immune surveillance role of CRT, our results showed that tmCRT/39-272 promoted tumorigenicity and there was a relative increase in M2 macrophages in the B16-tmCRT/39-272 cell and macrophage coculture system, indicating that tmCRT/39-272 improved macrophage polarization. Consistent with the *in vitro* results, M2 macrophages were also significantly enriched in the B16-tmCRT/39-272 tumors. Therefore, we conclude that tmCRT/39-272 may participate in M2 macrophage polarization.

To investigate the role of macrophages in the tmCRT/39-272-mediated stimulation of tumor development, we predepleted macrophages by i.p. injection of Lipo-Clod before inducing tumor formation. The tmCRT/39-272 effectively inhibited *in vivo* tumor formation in the mice predepleted of macrophages and subsequently reduced M2 macrophage polarization. According to our previous study, sCRT promoted the malignant progression of murine melanoma mainly through TLR4- and S100A8/9-mediated MDSC differentiation/generation and recruitment. TLR4 also acts as the receptor for cell surface CRT to mediate focal adhesion disassembly. Our results showed that tmCRT/39-272 might also interact with TLR4 to promote tumorigenesis. A body of evidence has shown that the immunosuppressive cytokine TGF-*β* plays a major role in blocking immune responses, affecting tumor development and progression. M2 macrophages promoted tumorigenesis by releasing large amounts of anti-inflammatory cytokines, such as TGF-*β* and IL-10 [44, 45]. In the present study, tmCRT/39-272 promoted Foxp3^+^ Treg infiltration in the tumor tissue by producing more TGF-*β* by stimulating M2 macrophages. The results were consistent with our *in vitro* study, where tmCRT/39-272 elevated TGF-*β* release, and the effects were reversed by anti-TGF-*β* antibody *in vitro.* Taken together, we conclude that tmCRT/39-272 activated the immune system and initiated the immune response at the early stage of tumor progression, and macrophages were activated and released a large amount of proinflammatory cytokines, contributing to the phagocytosis of tumor cells. With the development of tumor malignancy, tmCRT/39-272 promoted macrophage polarization to the M2 phenotype and the TGF-*β* secreted by M2 macrophages accelerated Treg differentiation in the TME, facilitating tumor growth and diffusion. Therefore, tmCRT/39-272 plays a major immunomodulatory role in tumor development and progression.

## Data Availability

The data used to support the findings of this study are available from the corresponding author upon request.

## Supplementary Materials

Supplementary Materials Supplementary Figure S1: proportions of MDSC/NK/DC/CD4^+^ T/CD8^+^ T cells remained comparable between B16-EGFP and B16-tmCRT/39-272 groups. Single cells were collected from tumor tissues of tumor bearing mice 18 days postinoculation, and stained with antibodies against CD45, Gr-1, CD11b, CD3, NK1.1, CD11c, CD4, or CD8 (in different combinations). The proportions of (a) MDSC (Gr-1 + CD11b+), (c) NK (CD3 + NK1.1+), (C) DC (CD11b + CD11c+), (d) CD4+ T (CD3 + CD4+), and (e) CD8+ T (CD3 + CD8+) were analyzed by FACS.
