# Supplementary material for: Membrane Bound CRT Fragment Accelerates Tumor Growth of Melanoma B16 Cell In Vivo through Promoting M2 Polarization via TLR4
Source: J Immunol Res. 2022 Oct 6;2022:4626813. doi: 10.1155/2022/4626813 (PMC9560857; doi:10.1155/2022/4626813)
Supplement: Supplementary Materials — Supplementary Figure S1: proportions of MDSC/NK/DC/CD4+ T/CD8+ T cells remained comparable between B16-EGFP and B16-tmCRT/39-272 groups. Single cells were collected from tumor tissues of tumor bearing mice 18 days postinoculation, and stained with antibodies against CD45, Gr-1, CD11b, CD3, NK1.1, CD11c, CD4, or CD8 (in different combinations). The proportions of (a) MDSC (Gr-1 + CD11b+), (c) NK (CD3 + NK1.1+), (C) DC (CD11b + CD11c+), (d) CD4+ T (CD3 + CD4+), and (e) CD8+ T (CD3 + CD8+) were analyzed by FACS. [file 4626813.f1.docx]

**Figure S1:**


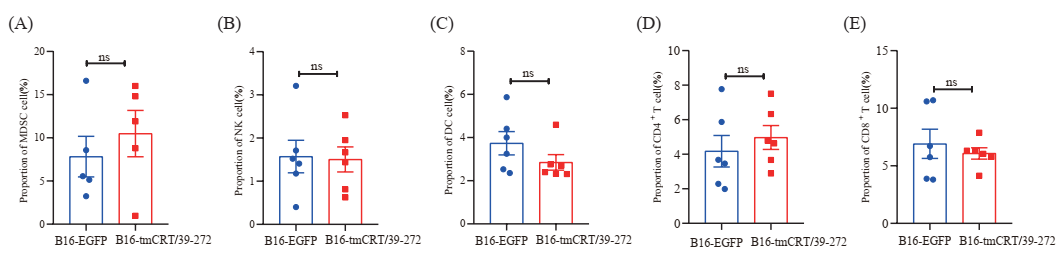


**Figure S1:** Proportions of MDSC/NK/DC/CD4^+^ T/CD8^+^ T cells remained comparable between B16-EGFP and B16-tmCRT/39-272 groups. Single cells were collected from tumor tissues of tumor bearing mice 18 days postinoculation, and stained with antibodies against CD45, Gr-1, CD11b, CD3, NK1.1, CD11c, CD4, or CD8 (in different combinations). The proportions of (a) MDSC (Gr-1^+^CD11b^+^), (b) NK (CD3^+^NK1.1^+^), (c) DC (CD11b^+^CD11c^+^), (d) CD4^+^ T (CD3^+^CD4^+^), and (e) CD8^+^ T (CD3^+^CD8^+^) were analyzed by FACS.
